# Effect of saturated and unsaturated fat on the physical properties of plant-based cheese

**DOI:** 10.1016/j.crfs.2024.100832

**Published:** 2024-08-30

**Authors:** Cameryn Sanders, Stacie Dobson, Alejandro G. Marangoni

**Affiliations:** Department of Food Science, University of Guelph, 50 Stone Rd E, Guelph, Ontario, Canada, N1G 2W1

**Keywords:** Plant protein, Texture, Rheology, coconut oil, Sunflower oil

## Abstract

In many plant-based meat and dairy alternatives, coconut oil is frequently used to replicate the textural and structural properties of animal fats due to its high saturated fat content. Concerns about the health implications of saturated fat and the sustainability of coconut oil call for an exploration into alternative fat combinations in plant-based foods. The effects of saturated fatty acid (SFA) content on plant-based cheese physical characteristics were evaluated through five different ratios of coconut oil (CO) to sunflower oil (SO): 100%, 90%, 75%, 60%, 50%, 40%, 25%, 10%, and 0%. As determined through texture profile analysis, the hardness of the cheeses after setting at 5°C for 24h increased with increasing amounts of coconut oil due to the increasing solid fat content providing additional firmness. The samples with 100% coconut oil displayed satisfactory melt and stretch; however, the melt and stretch values were matched by adding as little as 25% sunflower oil. The melt and stretch values did not continue to increase with increasing saturated fat content but instead remained constant with increasing coconut oil addition. Rheological analysis of the cheeses during a temperature ramp from 20 to 95°C was assessed where the tanδ value at 95°C was used as a measure of cheese melt, where values ≥ 1 indicated a better melt. The 0% coconut oil cheese had the lowest tanδ (G″/G′) value of 0.3, whereas the addition of 25% coconut oil into the cheese resulted in the tanδ increasing to values greater than 0.5. The 25% CO cheese sample also achieved a more similar complex viscosity (η*) to that of dairy cheese than all samples but the 75% CO cheese. Therefore, there is an opportunity to decrease the amount of coconut oil in plant-based cheese systems while maintaining good functional properties and improving the sustainability and health benefits of the final product.

## Highlights

• Dairy cheese is mimicked by plant-based cheese using plant protein and waxy starch.
• Starch gelatinization and retrogradation are modulated through heated mixing.
• New product with better melt, stretch, and nutrition than commercial products.
• Plant-based cheese can match the mechanical properties of conventional dairy cheese.

## Graphical abstract

**Figure:**
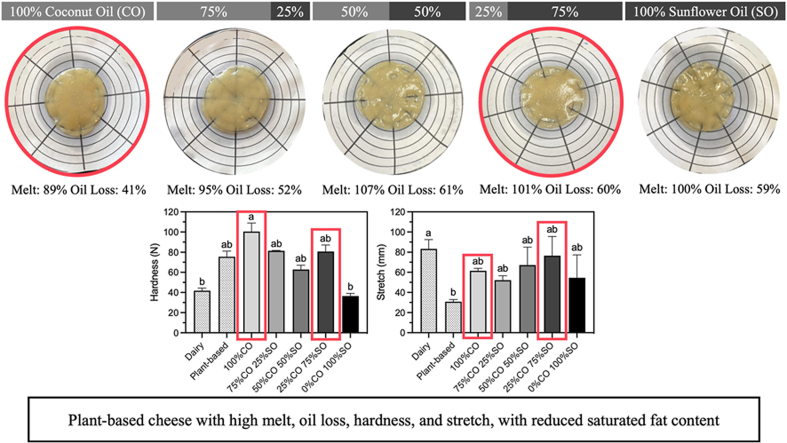

## 1 Introduction

Individuals may transition towards plant-based alternatives for numerous reasons (Ma et al., 2022). There are health, environmental, and animal welfare concerns that are increasing the popularity of plant-based alternatives (Bouvard et al., 2015; Godfray et al., 2018; Lonnie and Johnstone, 2020; Springmann et al., 2018). In fact, the environmental impacts of even the least environmentally demanding animal products are much greater than the average impact of vegetable proteins when considering greenhouse gas (GHG) emissions, eutrophication, acidification (excluding nuts), and land usage (Poore and Nemecek, 2018). A shift towards plant-based diets as opposed to meat and dairy shows promise for the reduction of GHG emissions, as well as for human health benefits, including nutritional value (Ferawati et al., 2021; Stehfest et al., 2009). As consumers make the transition from traditional dairy cheeses for any of these reasons, plant-based cheeses are forced to evolve. Similar to other plant-based analogues, plant-based cheese alternatives are expected to accurately mimic the taste, texture, functionality, and nutrition of dairy cheeses (Hanley et al., 2024; Kyriakopoulou et al., 2021). However, achieving these desirable characteristics involves many factors, prompting an exploration into the role of saturated and unsaturated fats.

Fat plays many roles in food products, influencing their appearance, texture, flavour, and nutritional value. In traditional dairy cheese, the naturally present fats contribute to meltability, creaminess, and desirable mouthfeel (De Bouillé, 2016). Recreating these characteristics in plant-based cheese partially depends on the 3D fat crystal network formed by triglycerides (McClements and Grossmann, 2021a). A common plant oil used in plant-based alternatives is palm oil. Despite the versatility and affordability of palm oil, there are many concerns regarding its production (Tan et al., 2009). Palm oil plantations are often grown at the cost of tropical forest deforestation as well as peatland destruction, which are important for global carbon balance and water retention from the wet season (Tan et al., 2009). Deforestation has threatened many species, from animals to plants, whose natural habitat of tropical forests is being destroyed (Tan et al., 2009). The environmental impact of palm oil calls for more sustainable practices and alternative ingredient sources to be used. Unfortunately, due to the fatty acid profile of palm oil, it is difficult to substitute it for alternative oils (Parsons et al., 2020).

Coconut and babassu oil are both tropical oils that have similar properties and can be used in place of palm oil (Parsons et al., 2020). Coconut and babassu oils are still high in SFAs, which has made them less acceptable to consumers due to the increased risk of cardiovascular disease and the negative impact on the nutritional value of the food (Parsons et al., 2020; Savva and Kafatos, 2015). The melting temperature and profile of the fat sources influence their functionality as well as the mouthfeel of the food product (De Bouillé, 2016). The melting temperatures for coconut and palm oil are 25°C and 35°C, respectively (Parsons et al., 2020). Because of the difference in melting points, coconut oil is not an ideal substitute for palm oil and may contribute to a different texture in plant-based foods, particularly cheese. The SFA contents of palm oil (49.3g/100g), coconut oil (86.5g/100g), and babassu oil (81.2g/100g) are quite high (Savva and Kafatos, 2015). SFAs help provide structure to the food matrix as well as higher oxidative stability (Yi et al., 2017). In contrast, unsaturated fats are liquid at room temperature, so they are more likely to leak out and do not contribute to the expected solidity of food products at low temperatures (McClements and Grossmann, 2021a; Yi et al., 2017). They are also more likely to become rancid during both processing and storage, making the use of unsaturated fats a challenge in plant-based foods (McClements and Grossmann, 2021b). However, many unsaturated vegetable oils such as canola (7.4g SFA/100g), soybean (15.7g SFA/100g), and sunflower (10.1g SFA/100g) have more positive effects on health, due mainly to the mono and polyunsaturated fats present, including ω-3 fatty acids (Saini and Keum, 2018).

While the amount of saturated versus unsaturated fats in the foods affects their nutritional value, the overall fat content is also important for the absorption of nutrients. Vitamins A, D, E, and K are fat-soluble, meaning their availability of fat in the food matrix impacts the nutritional content of the food product (Stevens, 2021). For these vitamins to be absorbed by the body, they require dietary fat; therefore, adequate amounts of fat need to be consumed to ensure effective absorption of fat-soluble vitamins. Despite health concerns, it is not feasible to eliminate fats from plant-based foods, as they aid in the promotion of overall health in plant-based diets.

The aim of this research was to explore the interconnection between saturated and unsaturated fats and their resulting impact on the functionality of plant-based cheeses. When creating an analogue to dairy cheese, the right balance of texture, meltability, and mouthfeel are only a few characteristics that need to be achieved. Previous research from our laboratory has produced a high-protein plant-based cheese formulation using waxy starch, plant protein, and coconut oil that achieved statistically similar hardness to a processed cheese single and commercial cheddar (Dobson and Marangoni, 2023). Using this as a baseline formulation, we have been able to modify the fat ratio to incorporate sunflower oil as a source of unsaturated fat in our plant-based cheese.

In this study, a range of cheeses with varying amounts of coconut and sunflower oil were evaluated and compared to an existing plant-based cheese as well as commercial dairy cheese. A sequence of analytical tests was employed to quantitatively and qualitatively assess the cheese products to summarize the ability to mitigate the undesirable environmental and health effects of coconut oil.

## 2 Materials and methods

### 2.1 Materials

Commercial dairy and non-dairy cheeses were procured from a local retailer. Black Diamond Mozzarella (Lactalis, Toronto, ON, CA). Daiya Classic Mozza Block (Daiya Foods Inc., Burnaby, BC, CA). Violife Mozzarella Style Shredded alternative to cheese (Arivia S.A, Thessaloniki, Greece). The pea protein isolate, waxy maize starch, and refined coconut oil were provided by a commercial supplier. 100% Pure sunflower oil (Kernel, Product of Ukraine. Manufactured for: Buzzfeed Tasty, New York, NY, USA) was bought from a local grocery store. Sodium chloride was purchased from Fisher Scientific (Ottawa, ON, CA). Lactic acid was purchased from Modernist Pantry LLC (Eliot, ME, USA).

### 2.2 Plant-based cheese methods

The formulation and mixing protocol were based on previously determined methods (Dobson and Marangoni, 2023).

#### 2.2.1 Formulation

Plant-based cheese was created using a formulation containing 7.5%w/w protein isolate, 21%w/w fat in the form of coconut oil, sunflower oil, or a blend of both, 21%w/w waxy corn starch, 0.8%w/w salt, and 49%w/w water. The mixture was adjusted to pH 5 using lactic acid. The formulation made with 21% coconut oil was used as a benchmark for functionality. Pea protein isolate was chosen as the primary protein based on its behaviour in the benchmark formulations, as it performed well in the system compared to other plant proteins.

#### 2.2.2 Mixing method

A 1%w/v protein solution using the protein ingredient and water was mixed on a stir plate at 400rpm for 10min or until mixed. The coconut oil was heated in the microwave to ensure it was completely melted before being added to the protein solution and homogenized at 20000rpm for 1min with a hand homogenizer. When sunflower oil was used in the formulation, microwaving was unnecessary as it was already liquid at room temperature. The protein, water, and oil mixture was poured into a Thermomix TM6 heated blender (Vorwerk & Co. KG, Wuppertal, Germany). While being mixed at 200rpm, the dry starch and remaining dry protein were added until the mixture was smooth. The salt was added, as well as the lactic acid to lower the pH to 5.0.

The mixture underwent heated mixing, following 5 steps.

1) Temperate ramp from 40°C to 80°C with mixing at 200rpm
2) Temperature hold at 80°C with speed ramp to 800rpm
3) Temperature hold at 80°C with mixing at 50rpm
4) Temperature hold at 80°C with speed ramp up to 800rpm
5) Temperature hold at 80°C with mixing at 50rpm

Samples were taken at various time points, identified as Time 4, Time 6, and Time 8. The cheese was refrigerated at 5°C for 24h before testing. For this study, only the Time 6 samples (which underwent heated mixing for 24min) were tested.

### 2.3 Methods for analyses

#### 2.3.1 Texture profile analysis

Texture profile analysis (TPA) was used to determine the sample hardness by compressing the sample by 50%. The texture profile analysis was based on previously determined methods (Dobson and Marangoni, 2023).

#### 2.3.2 Cheese shred efficacy

The blocks with a length of 50mm, a width of 25mm, and a height of10 mm were first weighed on a ME4002E precision balance (Mettler Toledo, Columbus, OH, USA) and the value was recorded in grams. The block was then placed into the manual drum grater (Starfrit, Atlantic Promotions Inc, Longueuil, QC, CA) and shredded until no more whole shreds fell from the drum. The weight of the shreds was then determined using the same balance and recorded in grams. The shred efficacy of the cheese was calculated by dividing the weight of the shreds by the original weight of the block, presented as a percentage. The shreds were placed into labelled, sealed plastic bags and kept at 5°C until further analysis.

#### 2.3.3 Cheese shred textural analysis

The shredded sample from the shred efficacy test was used to analyze the hardness of the shreds. The samples were kept at 5°C until analysis. The TA.XT2 texture analyzer (Stable Micro systems, Texture Technologies Corp. Scarsdale, NY, USA) was fitted with a 5kg load cell and a Warner Bratzler blade with the blade reversed so that the 1 mm flat edge was used. The blade was moved to a preset location of 5mm at 20mm/s. For analysis, a single shred was placed on the platform under the blade. The probe moved at 1.00mm/s for 4.750mm. The test was repeated for a minimum of 5 shreds. The data was recorded in newtons and analyzed using Exponent software.

#### 2.3.4 Modified schreiber disk melt test

The modified Schreiber melt test was used for a cheese sample after being heated at 232°C (450°F) for 5min. The melt was calculated based on the increase in spread that occurred across the concentric circles from the size of the sample prior to melting. This test was based on previously determined methods (Dobson and Marangoni, 2023).

#### 2.3.5 Oil loss

The amount of oil loss from the samples was measured based on the amount of oil saturation on the Schreiber disk paper from the disk melt test. The oil loss determination was based on previously determined methods (Dobson and Marangoni, 2023).

### 2.4 Rheology temperature sweep

Shear rheological measurements and temperature sweeps were performed using a rotational rheometer (MRC 302, Anton Paar, Graz, Austria) fit with a 20mm parallel plate geometry (PP20/S). To avoid slipping, the top and bottom plates were affixed with 40 and 600 grit sandpaper respectively. Peltier plates and a forced air hood (Anton Paar, Graz, Austria) were used to control the temperature.

2g of shredded cheese sample was weighed using a ME4002E precision balance (Mettler Toledo, Columbus, Ohio, USA). The shreds were then placed in a small circular mold 2.5 mm in diameter. The measuring system was lowered to 6mm to compress the shredded sample into a disk. The force was changed to 5N to further compress the sample to 1mm in height. Mineral oil was applied around the edges of the sample to prevent drying.

The test began with a temperature hold at 20°C at a normal force of 0.1N. The temperature was then ramped up to 95°C and carried out at 0.1% strain, at a frequency of 1Hz with a constant normal force of 0.1N to adjust for sample melting. The sample was then held at 95°C with 0.1% strain, a frequency of 1Hz, and a normal force of 0.1N.

#### 2.4.1 Axial pull

The stretch of the cheeses was measured using a rotational rheometer (MRC 302, Anton Paar, Graz, Austria) with Peltier plates and a forced air hood (Anton Paar, Graz, Austria) used for temperature control. To evaluate the melting profile of the cheeses, the variables obtained for the tests were storage modulus (G′), loss modulus (G″) and tan delta (G″/G′). After the heating ramp and hold were completed, an axial pull was performed where the top parallel plate geometry moved upwards at a speed of 1500um/s. Using RheoCompass Software, the Normal force (N) and Gap (mm) were recorded during the pull. A video recording using the camera of an iPhone 12 (Apple Inc.) captured the gap size of the instrument, the sample stretch, and the gap at which the sample broke, which was used as the break point. The final stretch was measured by Eq. (1):

(Eq.1) Stretch(mm)=Breakpoint(mm)−Gapatstartafterheating(mm)

#### 2.4.2 SFC melting profile and time curve for crystallization kinetics

The solid fat content (SFC) of the oil blends was measured using pulsed nuclear magnetic resonance (NMR) (minispec mqone SFC Analyzer, Bruker, Billerica, MA, USA). The samples were measured between 5°C and 30°C at 5°C intervals (AOCS Official Method Cd 16b-93) for the melting profile. For the time curve, the samples were measured at 5°C over 2h, beginning at 10s between tests and increasing gradually to intervals of 4 min until the end of the test. For both analyses, the samples were measured in standard NMR tubes of 180mm in height and 9mm in diameter. The Avrami model was fitted to the SFC-time data by non-linear regression in order determine crystallization kinetics, using Eq. (2) and GraphPad Prism 10.0 (GraphPad Software, San Diego, CA, USA) (Marangoni, 2017):

(Eq. 2) $$SFC={SFC}_{\max}\left(1-e^{{{-k}_{A}\left(X+t\right)}^{n}}\right)$$

The half-life of crystallization was determined using Eq. (3) (Marangoni, 2017):

(Eq. 3) $$t_{1/2}={\left(\ln\;2/k_{A\;}\right)}^{-n}$$

#### 2.4.3 Differential scanning calorimetry

The melting and cooling (crystallization) behaviour of the oil blends and oil blend cheese alternative samples were evaluated using differential scanning calorimetry (DSC 1 Star System, Mettler Toledo, Columbus, OH, USA). Approximately 20mg of sample was weighed into the aluminum pans before being hermetically sealed. The oil blends were cooled to −5°C and then melted to 80°C. The plant-based cheese samples were taken out of the 5°C refrigerator, then tested from 5°C to 120°C at the rate of 5°C/min. Peaks were then evaluated using STARe software 12.10 (Mettler-Toledo).

#### 2.4.4 X-ray diffraction (XRD)

Powder x-ray diffraction was performed using a Miniflex 600 6G Benchtop XRD (Rigaku, Tokyo, Japan). The XRD powder spectra were obtained on both the oil blends and the oil blend cheese alternatives. The oil blends were poured into the metal sample holders, then allowed to sit at 5°C for 24h before testing. The oil blend cheese alternatives were taken from the fridge at 5°C, then trimmed to fit into the sample holders and measured. For both types of samples, a 600W x-ray tube (Cu Kα/1.54 Å) was used to obtain spectra at 5°C from 2° to 50° 2θ, at a scan rate of 5°/min. The peak positions were determined using SmartLab Studio II software (Rigaku).

#### 2.4.5 Polarized light microscopy and fractal dimension

The fat microstructure was identified using polarized light microscopy through an OMAX M838PL-C180U3 (OMAX, Kent, WA, USA). The samples were melted at 80°C for 1h to erase crystal memory and 5 μL was transferred onto glass microscope slides with a coverslip placed on top. The samples were held at 5°C in the fridge for 24h before testing. During testing, the slides were removed from the fridge and promptly placed on a brass cold stage held at 5°C. Images were taken at 4x using ToupView software. The images were then imported into ImageJ software (Image analysis software, NIH, USA) where they were each converted to an 8-bit grayscale image, cropped to a 1.5mm × 1.5mm square from the centre of the image, and thresholded using the Moments setting. Fractal box counting was performed using ImageJ software. The box count value (D) for each sample was recorded.

### 2.5 Statistical analysis

Each set of samples was prepared in duplicate or more. Statistical analysis for all data was conducted using GraphPad Prism 10.0. Significance between samples (*p*<0.05) was assessed through one-way ANOVA followed by Tukey's Multiple Comparison Test.

## 3 Results and discussion

### 3.1 Functional characteristics of commercial dairy, commercial plant-based cheese, and plant-based cheeses made with coconut and sunflower oil blends

**Fig. 1:**
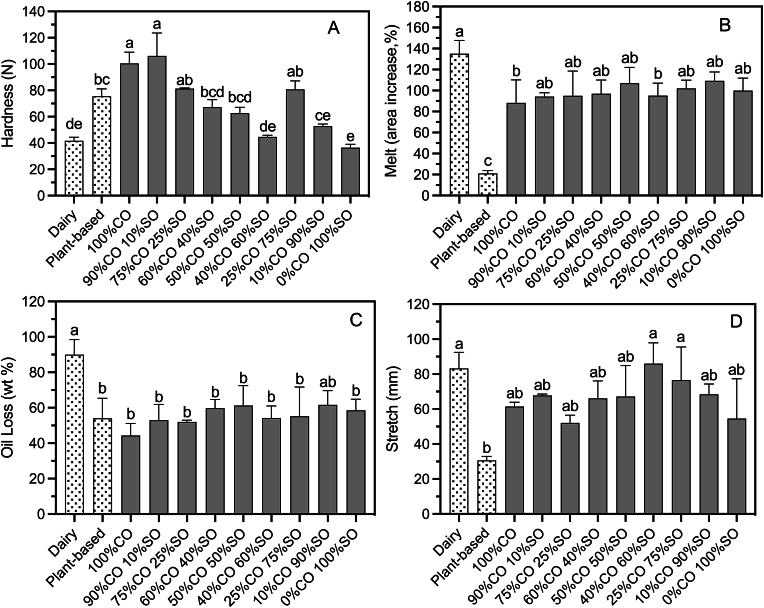
Functional properties of a commercial dairy mozzarella, a commercial plant-based mozzarella alternative, and CO-SO blend plant-based cheese alternatives; A) TPA Hardness (N), B) Melt (area increase, %), C) Oil Loss (wt %), and D) Stretch (mm). Values are mean (n≥3) ± standard deviation. Mean values labelled with different lower-case letters (a–e) are statistically different (*p*<0.05).

Fig. 1 below displays the functional properties of the dairy and plant-based cheeses that were evaluated. The first test in the table is hardness, which is a result of TPA. For this research, the aim was to produce a plant-based cheese of a hardness close to 80N to match a current commercial plant-based product. This is a higher hardness than a typical commercial mozzarella to ensure the product can be shredded and sliced efficiently so that minimal product is discarded during manufacturing. Therefore, the aim was to match the hardness of the 100% coconut oil formulation by replacing a portion of the coconut oil with sunflower oil. The oil blend cheese alternatives had hardness values ranging from 44N to 106N, where as much as a 75% sunflower oil substitution in the cheese was able to produce a statistically similar hardness to the commercial product. It was observed that the hardness of the cheese increased with increasing coconut oil content, except for the 25% CO blend cheese. Despite its minimal saturated fat content, the 25% CO sample displayed an unexpectedly high hardness that was more similar to the 100% CO cheese. The increasing hardness with increasing saturated fat trend was also observed in the shred hardness (Supplementary Table 1). The hardness also appeared to impact the shred efficacy values (Supplementary Table 1). Shred efficacy represents how much of the sample would be discarded after being shredded. Only the percentage of shreds recovered from the grater can be packaged. Cheeses that were softer would begin to adhere to the sides of the grater, reducing the weight of the shreds produced. Overall, the 60% CO, 40% CO, 10% CO, and 0% CO oil blend cheese alternatives reached TPA hardness values statistically similar to commercial dairy mozzarella, proving that if desired, the oil blend cheese alternatives can be used in a range of food applications.

When consuming dairy and plant-based cheeses, melt is one of the most sought-after characteristics. The commercial plant-based mozzarella style displayed a significantly poorer melt than commercial dairy mozzarella. On the other hand, the oil blend cheese alternatives, excluding the 100% CO and 40% CO blends, were able to achieve a statistically similar melt to the commercial dairy mozzarella after heating. Attaining a high melt in plant-based cheese is much more difficult than in dairy cheese, as dairy cheeses contain casein. The interactions of casein molecules with other casein molecules and water in the cheese are impacted when heated, disrupting hydrogen bonds, and resulting in melting (Atik and Huppertz, 2023; Ladjal-Ettoumi et al., 2016). When heated, the casein network contracts, reducing the number of crosslinks among casein micelles (Gonçalves and Cardarelli, 2021; Joshi et al., 2004). Since plant-based dairy alternatives do not contain casein, the melt must be achieved in another way, so the focus is on the fat, protein, and starch present. Dobson and Marangoni (2023) noted that high amylopectin starches provide better softening than high amylose starches and, therefore, better melting in plant-based cheese (Dobson and Marangoni, 2023). This technology was used to substitute up to 90% SO for CO and reach a statistically significant melt to the 100% CO cheese. Since the melt was mostly provided by starch retrogradation, the substitution of saturated to unsaturated fat did not appear to have a significant effect on the melt.

However, the oil loss of the samples is more closely related to the fat. Oil loss represents the amount of oil that has been expelled from the food matrix during heating. Where oil loss is anticipated in dairy cheese due to the melting of saturated fat, oil loss in plant-based cheese is not guaranteed. Oil loss in plant-based cheeses can have adverse effects on their application, but it can also be beneficial in terms of physical characteristics. Some free oil formation is important to the melting of the cheese in a practical application (Everett and Auty, 2008; Mattice and Marangoni, 2020). Commercial dairy cheese has a high oil loss at 90%, which may be undesirable on a pizza, for example, as it can create unappealing pools of oil on the surface. On the other hand, too little oil loss, as seen in the 100% CO cheese at 41% oil loss, correlates with less lubricity in the cheese, which is not ideal for texture and mouthfeel. The unsaturated oil contributed to solving this, as it was more likely to leak out during heating than coconut oil. However, there were no significant differences between the oil losses across the oil blend cheese alternatives.

For dairy mozzarella cheese to become flowable and elastic, the protein matrix must be disrupted and weakened (Johnson, 2000; Mohd Shukri et al., 2022). Depending on the strength of the hydrophobic interactions, some proteins may not be disrupted and, therefore, unfold, which affects the flow of cheese (Paulson et al., 1998). To ensure a complete melt before extension, the cheese samples were heated to 95°C. The stretch values of the oil blend cheese alternatives were all greater than the stretch of Daiya Mozzarella Style, showing that the disruption of the protein network benefits the extensibility of the cheese, as most commercial plant-based cheese alternatives contain little to no protein. However, if too much energy is required to disrupt the network during the heating period, the cheese will not melt or stretch as well. In addition, the melting of fat in the cheese also helps with its ability to flow (Paulson et al., 1998). The oil blend cheese alternatives were still able to achieve statistically similar stretch values to commercial dairy mozzarella, despite containing varying amounts of saturated fat. The fat globules present in the network melt when heated, which disrupts the protein network, allowing for the starch to enable stretch (Dobson and Marangoni, 2023). There was little variance in stretch values across the oil blend cheese samples. Commercial dairy mozzarella reached a long stretch value of 83mm, while Daiya Mozzarella Style only reached a 31mm extension. The low melt of the Daiya Mozzarella Style correlates with its minimal stretch due to poor softening. On the other hand, the stretches of the oil blend cheese alternatives, along with the commercial dairy mozzarella, were much more fluid and cohesive, which was consistent with their high melt and spreadability.

### 3.2 Rheological melting characteristics of commercial dairy, commercial plant-based cheese, and plant-based cheeses made with coconut and sunflower oil blends

**Fig. 2:**
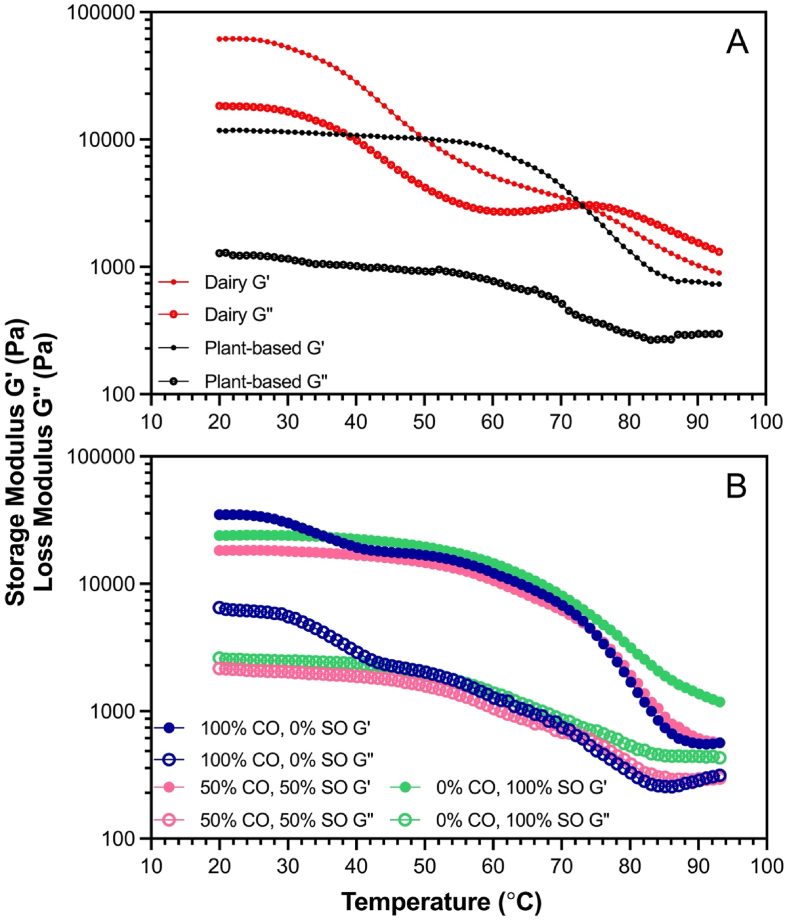
Rheological melting profiles displaying Storage Modulus (G′) and Loss Modulus (G″) over temperatures from 20 to 95°C, for A) Commercial Mozzarella and Daiya Mozzarella Style, B) 100% CO 0% SO, 50% CO 50% SO, and 0% CO 100% SO oil blends. Values are the mean n*≥*3.

The rheological properties of commercial mozzarella, commercial plant-based, and oil-blend cheeses are presented in Table 1. Commercial dairy mozzarella cheese exhibits more solid behaviour at 20°C, where the storage modulus (G′) is greater than the loss modulus (G″). The crossover point where G′ is equal to G″ occurs at around 73°C as the cheese becomes increasingly more viscous, until G″ is greater than G′. The tanδ (G″/G′) represents this change that has occurred, where the tanδ value approaching one or greater indicates a more desirable melt. With the commercial dairy mozzarella in Fig. 2A and the 100% CO blend cheese in Fig. 2B, there is a notable decrease in value from 20°C to 40°C. This decrease is indicative of the initial saturated fat melting in the sample; in the dairy mozzarella, it is milk fat, and in the 100% CO blend, it is coconut oil. This decrease becomes less noticeable as the amount of unsaturated oil in the sample increases, as seen in Fig. 2B with the 50% CO and 0% CO oil blend cheese alternatives. These two samples have a plateau until around 70°C.

**Table 1:** Shear rheological parameters at 40°C and 95°C for commercial dairy mozzarella, a commercial plant-based mozzarella alternative, and coconut and sunflower oil blend plant-based cheese alternatives.

Sample	G′_40°C_ (Pa)	G″_40°C_ (Pa)	tan***δ***_40°C_ (−)	G′_95°C_ (Pa)	G″_95°C_ (Pa)	tan***δ***_95°C_ (−)	η*_95°C_ (Pa s)
*Dairy*							
Black Diamond Mozzarella	32169 ± 10520^a^	11159 ± 3576^a^	0.35 ± 0.01^a^	583.1 ± 409.0^a^	1111 ± 258.9^a^	1.42 ± 0.05^a^	216 ± 48.2^a^
*Plant-Based*							
Daiya Mozzarella Style	10744 ± 1451^b^	1015 ± 154.4^b^	0.10 ± 0.01^c^	728.6 ± 189.8^a^	304.9 ± 29.90^b^	0.43 ± 0.06^b^	126 ± 29.8^ab^
*Coconut and Sunflower Oil Blends*							
100% CO, 0% SO	17687 ± 627.5^ab^	2611 ± 396.2^b^	0.14 ± 0.01^b^	677.6 ± 21.74^a^	394.6 ± 18.52^b^	0.58 ± 0.02^b^	125 ± 4.26^ab^
90% CO, 10% SO	14896 ± 11176^ab^	2765 ± 645.3^b^	0.13 ± 0.02^bc^	669.8 ± 83.50^a^	380.9 ± 15.09^b^	0.43 ± 0.05^b^	123 ± 12.7^ab^
75% CO, 25% SO	24899 ± 9523^ab^	3227 ± 2005^b^	0.13 ± 0.03^bc^	1064 ± 762.4^a^	456.5 ± 140.0^b^	0.51 ± 0.17^b^	116 ± 12.3^ab^
60% CO, 40% SO	22028 ± 5711^ab^	2486 ± 630.8^b^	0.11 ± 0.01^bc^	697.0 ± 22.59^a^	389.9 ± 18.78^b^	0.59 ± 0.07^b^	127 ± 4.51^ab^
50% CO, 50% SO	16715 ± 3209^ab^	1872 ± 244.4^b^	0.11 ± 0.01^bc^	559.6 ± 164.2^a^	311.0 ± 76.98^b^	0.56 ± 0.03^b^	102 ± 28.8^b^
40% CO, 60% SO	23676 ± 776.9^ab^	2664 ± 277.7^b^	0.11 ± 0.02^bc^	644.3 ± 82.39^a^	375.9 ± 36.98^b^	0.59 ± 0.03^b^	119 ± 14.1^ab^
25% CO, 75% SO	20314 ± 1979^ab^	2210 ± 560.4^b^	0.11 ± 0.02^bc^	840.2 ± 163.6^a^	462.2 ± 55.10^b^	0.54 ± 0.04^b^	153 ± 27.0^ab^
10% CO, 90% SO	19068 ± 4828^b^	1992 ± 575.0^b^	0.10 ± 0.01^bc^	628.9 ± 189.2^a^	369.4 ± 92.17^b^	0.59 ± 0.04^b^	116 ± 33.4^ab^
0% CO, 100% SO	22396 ± 2371^b^	2386 ± 348.4^b^	0.11 ± 0.02^bc^	712.3 ± 141.1^a^	389.0 ± 46.29^b^	0.45 ± 0.06^b^	129 ± 22.8^ab^

Values are mean (n≥3) ± standard deviation. Values followed by different superscript letters (a-c) within the same column are statistically different (*p*<0.05).

There is a second notable decrease in value at 68°C for the Daiya Mozzarella Style in Fig. 2A and at 70°C for the oil blend cheese alternatives seen in Fig. 2B. This decrease is associated with the gelatinization of the waxy starch, resulting in slight softening of the sample (Dobson and Marangoni, 2023). The G′ of the commercial mozzarella cheese has a more consistent decrease until the final temperature of 95°C, which doesn't include a plateau like that is seen with the plant-based cheese alternatives. The G″, however, slightly plateaus around 62°C, until the crossover point where it decreases more rapidly, around 74°C. The tanδ values of the plant-based samples are significantly higher at 95°C compared to 40°C as supported in Table 1. At 40°C, the commercial dairy mozzarella has a significantly larger tanδ value at 0.35, over twice as great as the next largest value, which is the 100% CO blend at 0.14.

From these results, it was found that the tanδ values at 40°C and 95°C are not completely representative of the melt of the cheeses, as the tanδ values of the fat blend cheeses showed very little variance between samples. The complex viscosity ($\eta^{*}=\frac{G^{*}}{\omega }=\sqrt{\frac{{\left(G^{\prime }\right)}^{2}+{\left(G^{\prime \prime }\right)}^{2}}{\omega }}$) at 95 °C from G′ and G″ data, as well as the frequency of oscillation of 6.28rad/s from our experiments was therefore used to better understand the melt of the system. From this, it was shown that the 25% CO cheese sample can achieve a more similar complex viscosity (η*) to that of dairy cheese than all samples but the 75% CO cheese. This proves to be a more accurate approach to compare the functionalities of plant-based and dairy cheese in their molten state (95°C).

### 3.3 Solid fat content and crystallization kinetics

**Fig. 3:**
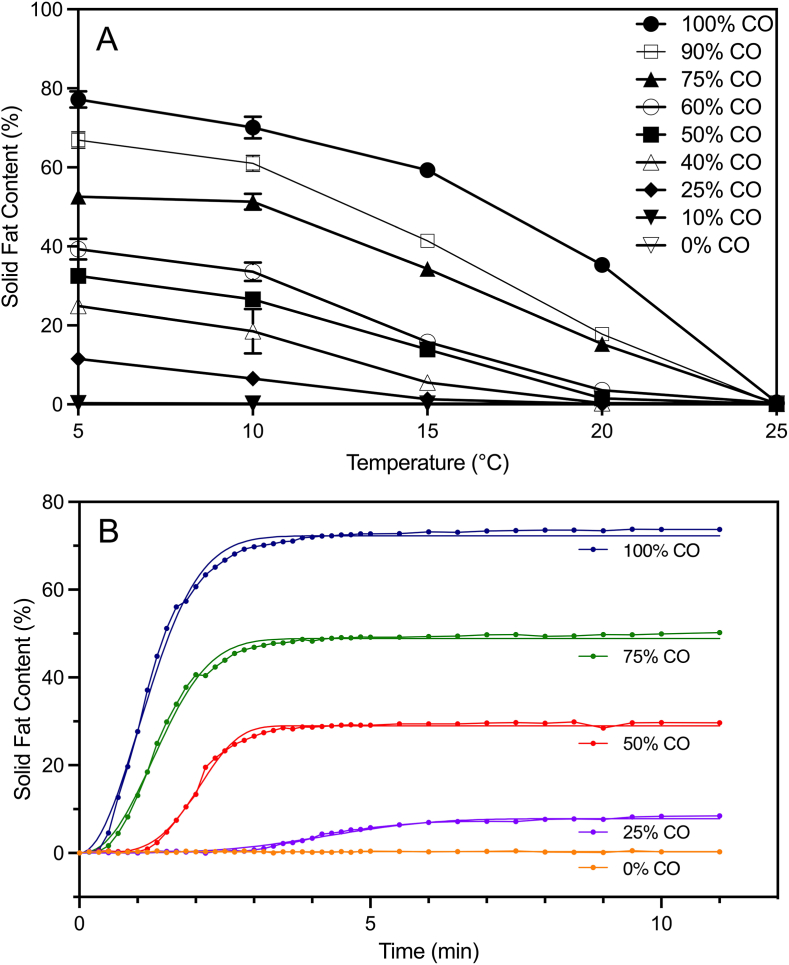
A) Solid fat content of the different CO-SO blends as a function of temperature, B) Avrami analysis fits (solid lines) of the crystallization curves (symbols) for the different CO-SO blends at 5°C. Values are mean (n≥2) ± standard deviation (error bars).

The change in SFC over temperature and time is heavily dependent on the coconut oil concentration, which provides most of the solid fat in the blends. With increasing proportions of coconut oil, the SFC increases as expected, as seen in Fig. 3A. At increasing temperatures, the SFC decreases as the temperature increases beyond the melting point of the coconut oil. The SFCs at both 5°C and 10°C follow a similar trend across the oil blends. There is no SFC observed for the 0% CO and 10% CO blends. It is not until the 25% CO ratio that the samples have an SFC of 5%–10%. The SFCs at 5°C and 10°C consistently increase until final values of 77.2% and 70.1%, respectively, at 100% CO. On the other hand, the SFC at 20°C remains below 5% until the 60% CO blend, where it increases to reach a final value of 35.3% for the 100% CO sample.

In Fig. 3B, the SFC had a relatively steep increase for both the 100% CO and 75% CO blends until around 120s, where they reached a plateau up to 75% and 50% SFC, respectively. The 50% CO blend had a gentler increase in SFC, not beginning to crystallize until 90s. This blend only reached a final SFC of 30%. The 25% CO oil blend shows no SFC until 200s, where the solid fat begins to crystallize, and the blend reaches up to 9% SFC around 300s. Consistent with Fig. 3A, no SFC is observed in the 0% CO oil blend. A similar melting behaviour relative to each sample is seen at all temperatures, proving how great of an effect the coconut oil has on the melting in cheese alternatives at increasing temperatures.

The crystallization kinetics of the CO-SO samples were quantified using half-life data calculated from values derived from the Avrami model. This model was fitted to the SFC crystallization curve data (Fig. 3B). The crystallization rates of the samples increased with increasing saturated fat content, as expected. However, as seen in Table 2, the 25%, 75%, and 100% CO samples had the most similar and longest half-lives, despite the 25% CO sample having a significantly lower SFC_max_. The Avrami index of 25% CO was dissimilar to the other samples, suggesting that it forms a different crystal structure. A lower $k_{A}$ and a larger n are characteristic of slower nucleation, possibly allowing the 25% CO blend to exhibit more nucleation sites, as well as more favourable polymorphic forms that enable it to reach a similar hardness to pure coconut oil.

**Table 2:** Avrami rate constant ($k_{A}$), Avrami index (n), half-life of crystallization ($t_{1/2}$), maximum solid fat content (SFC_max_), and fractal dimension values for the different CO-SO blends.

	100% CO	75% CO	50% CO	25% CO	0% CO
$10^{2}k_{A}$ (min^-n^)	48.43	33.52	3.628	0.5081	NC
n	2.0	2.3	4.2	3.4	NC
$t_{1/2}$ (min)	0.192	0.179	0.060	0.199	NC
SFC_max_ (%)	73.4	49.7	29.2	6.26	NC
Fractal Dimension	1.80	1.66	1.67	1.75	NC

### 3.4 Microstructure

**Fig. 4:**
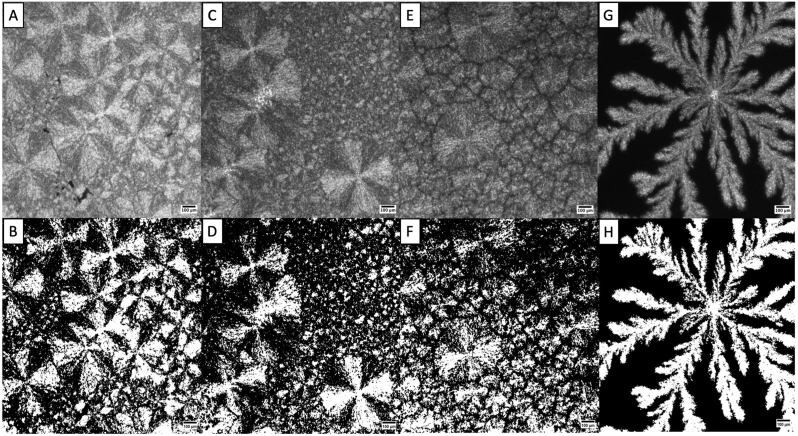
Polarized light micrographs of coconut oil and sunflower oil blends. A) grayscale image of 100% CO, 0% SO. B) thresholded image of A). C) grayscale image of 75% CO, 25% SO. D) thresholded image of C). E) grayscale image of 50% CO, 50% SO. F) thresholded image of E). G) grayscale image of 25% CO, 75% SO. H) thresholded image of G). Images were taken at 4X magnification, and the scale bar represents 100 μm.

The microstructure of the fat and oil blends gives insight into their impact on the overall cheese structure through the crystal networks of the fats, characterized by their respective measures of fractal dimension that are influenced by various structural factors (Tang and Marangoni, 2006a, 2006b). The fractal dimension values of the 100% CO, 75% CO, 50% CO, and 25% CO samples were determined using the box-counting method (Table 2). These values are used to investigate how the crystalline mass is organized spatially in each fat blend (Nicholson and Marangoni, 2020). Fig. 4 shows the grayscale and thresholded images of the microstructures observed under polarized light microscopy for each fat blend. Fig. 4A of the 100% CO samples has a more homogenous network of fat crystals, with a higher fractal dimension than the 75% and 50% CO blend samples. The 75% CO blend sample has a more visible separation of crystalline species, whereas the 50% blend sample displays a very strong grain boundary interface, as seen in Fig. 4E. In this sample, the crystals are growing into each other, excluding the liquid oil, which weakens the overall structure, resulting in a softer cheese. The 50% CO cheese sample displayed a hardness value of 63N from the textural profile analysis, which was lower than the hardness of the 25% CO cheese sample of 81N. This crystalline structure does not appear to affect the other functional properties of the cheese, as the melt, oil loss, and stretch values of the fat blend samples remain similar (Fig. 1).

The 25% CO blend sample (D = 1.75) from microscopy had a more similar fractal dimension to the 100% CO sample (D = 1.80) than either the 75% or 50% CO blends. This correlates with the Avrami indices from Table 2, where the 25% CO sample has an index that varies from the other samples. In addition, this sample also has a much different crystal structure than the other samples. Where the samples that contain 50%, 75%, and 100% CO appear to form Maltese crosses on the spherulites, the 25% CO sample forms a larger unique spherulite that is unlike the other samples. The increased amount of SO may be forcing the CO into crystallizing into a different form than when there is less SO present.

As the D value approaches 2, it becomes more indicative of a homogenous crystal network that is more clustered than it is distributed, based on the crystal distributions in space as well as their morphology (Nicholson and Marangoni, 2020; Tang and Marangoni, 2006a; Yadav et al., 2010). On the other hand, the 75% and 50% CO blends had lower fractal dimensions of 1.66 and 1.67, respectively, showing less dense networks. In most systems containing fats, textural properties such as hardness are dictated by the solid fat portion, however, these findings suggest that the TPA hardness of the cheese is not determined by the SFC of the fat blends but instead, by the fat microstructure, as seen in Fig. 4 (Tang and Marangoni, 2008).

### 3.5 Differential scanning calorimetry

**Fig. 5:**
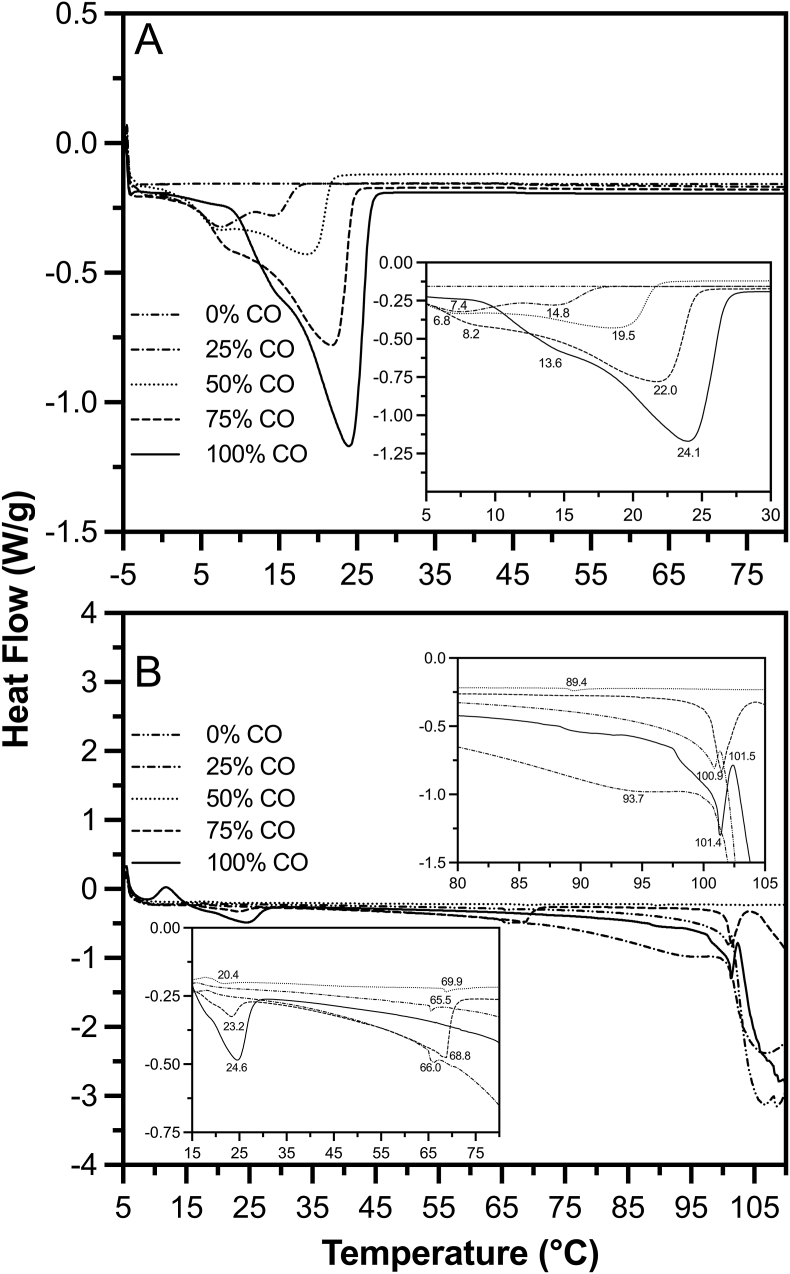
Differential Scanning Calorimetry melting profiles of CO-SO blends; A) CO-SO blends, B) CO-SO blends in cheese.

Fig. 5A depicts the melting profiles of the coconut and sunflower oil blends, without the presence of water, starch, or protein. This allows the differences between samples to be seen more clearly, based on the effect of SFC and saturated fat versus unsaturated oil on the melting of the samples. The coconut oil melting peaks are seen around 15°C–24°C, which correlates with the DSC results of the oil blend cheese alternative samples in Fig. 5B, where these peaks are seen around 21°C–25°C. There are two peaks for the oil blend samples containing CO, indicating different fractions of coconut oil melting at different temperatures. Fig. 5B shows the coconut oil peak begins around 25°C in the 100% CO oil blend cheese. As the percentage of CO decreases, the melting point is affected and the sample melts quicker, as shown in Fig. 5B, until there is no noticeable peak for coconut oil. There is no apparent coconut oil peak once the concentration drops below 50% CO, which correlates with Fig. 3A, showing that there is minimal SFC in the 25% CO and, of course, the 0% CO oil blend at 20°C. Within the fat blend samples in Fig. 5A, there are varying onset temperatures that occur. Overall, the addition of sunflower oil appears to decrease the melting temperature of coconut oil, especially in the second peak. This is important to note for food applications, as the melting point of the fat can greatly affect the textural properties of the product. If the fat in the cheese melts quickly and early into heating, the cheese may spread more, but this also increases the risk of the cheese burning or getting overly crisp. However, if the fat takes longer to melt, the cheese will likely melt more evenly, maintaining the fluidity of the cheese. The addition of unsaturated oil visibly impacts the melting temperature of the coconut oil and, therefore, the melting of the cheeses.

With a decrease in coconut oil in the samples, peaks associated with the gelatinization of waxy corn starch were observed. Typically, this peak is visible around 71.6°C–73.9°C (Paredes-López et al., 1994; Wang and Shi, 2020). However, the interactions within the cheese matrix caused a shift in this peak, now appearing at lower temperatures around 65°C–69°C. There was no starch peak in the 100% CO sample, possibly because the concentration of saturated fat inhibits the starch's ability to gelatinize, and the sample is left with ungelatinized starch.

The fat blend cheese samples showed peaks representative of protein, ranging from 89°C to 102°C, within the range of typical pea protein gelatinization that occurs around 83°C or 104°C (Ladjal-Ettoumi et al., 2016). Since there is a 49%w/w water content in the samples, the water peaks, also observed around 100°C, often coincide with those of the protein, making them difficult to differentiate. However, from these peaks, it is evident that this is a thermally stable protein with a higher denaturation temperature (Ladjal-Ettoumi et al., 2016; C.-H. Tang and Sun, 2011). A higher denaturation temperature may affect the cheese's ability to melt, as the sample requires more heating to break the hydrogen bonds, helping the cheese to flow.

### 3.6 X-ray diffraction

**Fig. 6:**
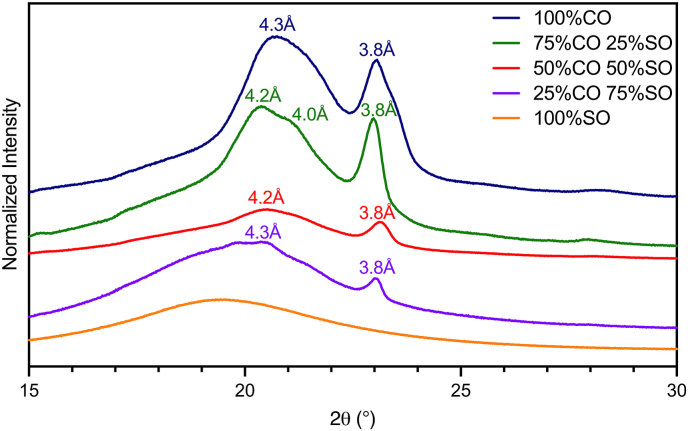
Powder X-ray diffraction spectra of coconut oil and sunflower oil blends; A) 100% CO, 0% SO, B) 75% CO, 25% SO, C) 50% CO, 50% SO, D) 25% CO, 75% SO, E) 0% CO, 100% SO.

**Fig. 7:**
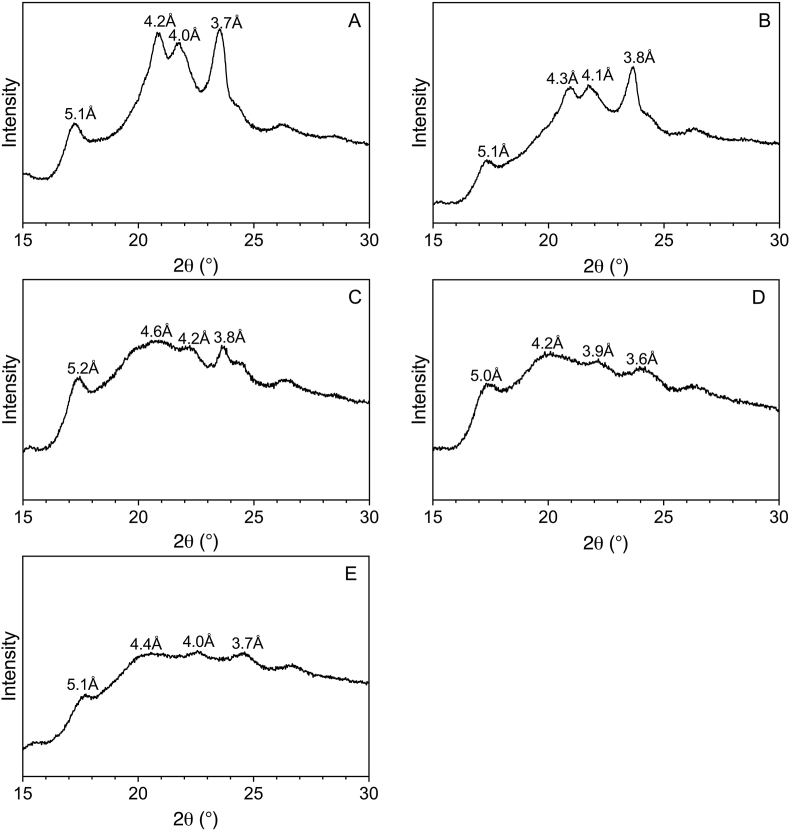
Powder X-ray diffraction spectra of cheese alternatives made with; A) 100% CO, 0% SO, B) 75% CO, 25% SO, C) 50% CO, 50% SO, D) 25% CO, 75% SO, E) 0% CO, 100% SO.

Depending on the polymorph in which the coconut oil is present, the stability and melting vary (Timms, 1984). The three polymorphs include α, β, and β′ (Timms, 1984). The β′ polymorphic form of coconut oil is typically observed in short-spacing (wide angle) regions of 3.8 Å and 4.2 Å, which can be seen in the spectra in Fig. 6 of the oil blends (Fredrick et al., 2008; Sonwai et al., 2016). Interestingly, the first peak of both the 100% and 25% CO blend spectra are slightly shifted, agreeing with the crystallization results from Fig. 4 and, therefore, the functional similarities between these samples. When the same oil blends are tested in the cheese alternatives (Fig. 7A–E), the α form at 4.1 Å is present, although slightly shifted, suggesting that potentially a different fraction of the fat is crystallizing into this form. This suggests the fat is more unstable when in the cheese system compared to its natural state, making it more susceptible to leaking out of the system to contribute to oil loss. The α form is the least stable of the three forms, with the lowest melting ability (Timms, 1984). The β form, however, is not observed, though it is the most stable form and exhibits the highest melting behaviour (Timms, 1984). Additionally, in the spectra of the cheese alternatives, a peak characteristic of amylopectin in the retrograded starch is visible around 5.1 Å. The last peak varies from 3.6 Å to 3.8 Å, as the 3.8 Å $\beta^{\prime }$ peak coincides with starch around the same *d* spacing, affecting the position and intensity of the resulting peaks. The strength of this peak decreases with decreasing CO, becoming more amorphous. With less saturated fat, the hardness resulting from the crystallization within the cheeses is more influenced by the retrograded amylopectin.

## 4 Conclusion

The sustainability and health of plant-based cheese alternatives have the potential to be improved through the implementation of alternative fat solutions. The plant-based cheese sector seems to currently rely heavily on coconut oil due to its ability to mimic the functional properties of animal fats. Therefore, a challenge arises due to the cultivation and processing of coconut oil, along with its high saturated fat content. Through the evaluation of a range of coconut oil to sunflower oil ratios in a novel plant-based cheese formulation, the hardness of the cheese was observed to typically increase with increasing coconut oil percentage, whereas the fractal dimensions did not support this trend. Therefore, saturated fat plays a role in structuring the cheese, but the textural properties were more influenced by the resulting fat microstructure. However, the reliance on coconut oil can be reduced in these samples by replacing as much as 75% of the coconut oil with sunflower oil without compromising the ideal hardness, melt, and stretch properties. The rheological melting profiles of the oil blend cheese alternatives were not able to match that of conventional mozzarella. However, the oil blend cheese alternatives surpassed the baseline plant-based cheese alternatives, proving that there is potential for growth in this area. Reformulating plant-based cheese alternatives to improve the fat profile can create opportunities to address common concerns surrounding unfamiliar food products, including health and sustainability. Further efforts are required to ensure the industry can match the expectations and preferences of consumers.

## CRediT authorship contribution statement

Cameryn Sanders: Validation, Formal analysis, Investigation, Writing – Original draft, Writing – review and editing, Visualization. Stacie Dobson: Conceptualization, Methodology, Validation, Formal analysis, Supervision. Alejandro G. Marangoni: Conceptualization, Methodology, Resources, Investigation, Data curation, Writing – review and editing, Supervision, Project administration, Funding acquisition.

## Declaration of competing interest

The authors declare that they have no known competing financial interests or personal relationships that could have appeared to influence the work reported in this paper.

## Data Availability

Data will be made available on request.
