# Supplementary material for: Effect of saturated and unsaturated fat on the physical properties of plant-based cheese
Source: Curr Res Food Sci. 2024 Aug 30;9:100832. doi: 10.1016/j.crfs.2024.100832 (PMC11403416; doi:10.1016/j.crfs.2024.100832)
Supplement: Multimedia component 1 [file mmc1.docx]

# Appendix A. Supplementary Data

The following are supplementary data to this article:

Supplementary Table 1. Functional properties of a commercial dairy mozzarella, a commercial plant-based mozzarella alternative, and coconut and sunflower oil blend plant-based cheese alternatives.

| **Sample** | **Shred efficacy (%)** | **Shred hardness (N)** |
| --- | --- | --- |
| *Dairy* |  |  |
| Black Diamond Mozzarella | 71±1^a^ | 0.68±0.1^b^ |
| *Plant-Based* |  |  |
| Daiya Mozzarella Style | 71±4^a^ | 1.48±0.1^a^ |
| *SO-CO blends* | | |
| 100% CO, 0% SO | 72±2^a^ | 0.92±0.2^b^ |
| 90% CO, 10% SO | 70±2^a^ | 0.85±0.4^bc^ |
| 75% CO, 25% SO | 73±3^a^ | 0.78±0.3^bc^ |
| 60% CO, 40% SO | 71±5^a^ | 0.59±0.2^bc^ |
| 50% CO, 50% SO | 73±5^a^ | 0.83±0.3^bc^ |
| 40% CO, 60% SO | 72±6^a^ | 0.47±0.3^c^ |
| 25% CO, 75% SO | 75±2^a^ | 0.49±0.4^c^ |
| 10% CO, 90% SO | 73±2^a^ | 0.69±0.2^bc^ |
| 0% CO, 100% SO | 72±4^a^ | 0.68±0.1^bc^ |

Values are mean (n≥3) ± standard deviation. Values followed by different superscript letters (a-c) within the same column are statistically different (*p*<0.05).
